# Notch and Wnt/β-catenin signaling pathway play important roles in activating liver cancer stem cells

**DOI:** 10.18632/oncotarget.6805

**Published:** 2015-12-31

**Authors:** Ronghua Wang, Qian Sun, Peng Wang, Man Liu, Si Xiong, Jing Luo, Hai Huang, Qiang Du, David A. Geller, Bin Cheng

**Affiliations:** ^1^ Department of Gastroenterology and Hepatology, Tongji Hospital, Tongji Medical College, Huazhong University of Science and Technology, Wuhan, China; ^2^ Department of Surgery, Starzl Transplantation Institute, University of Pittsburgh School of Medicine, Pittsburgh, Pennsylvania, USA

**Keywords:** hepatocellular carcinoma, cancer stem cells, Notch, Wnt/β-catenin

## Abstract

Human hepatocellular carcinoma (HCC) is driven and maintained by liver cancer stem cells (LCSCs) that display stem cell properties. These LCSCs are promoted by the intersecting of Notch and Wnt/β-Catenin signaling pathways. In this study, we demonstrate that LCSCs with markers CD90, CD24, CD13, and CD133 possess stem properties of self-renewal and tumorigenicity in NOD/SCID mice. The increased expression of these markers was correlated with advanced disease stage, larger tumors, and worse overall survival in 61 HCC cases. We also found that both Notch and Wnt/β-catenin signaling pathways played important roles in increasing the stem-ness characteristics of LCSCs. Our data suggested that Notch1 was downstream of Wnt/β-catenin. The active form of Notch1 intracellular domain (NICD) expression depended on Wnt/β-catenin pathway activation. Moreover, Notch1 negatively contributed to Wnt/β-catenin signaling modulation. Knock down of Notch1 with lentivirus N1ShRNA up-regulated the active form of β-catenin. Ectopic expression of NICD with LV-Notch1 in LCSCs attenuated β-catenin/TCF dependent luciferase activity significantly. In addition, there was a non-proteasome mediated feedback loop between Notch1 and Wnt/β-catenin signaling in LCSCs. The central role of Notch and the Wnt/β-catenin signaling pathway in LCSCs may provide an attractive therapeutic strategy against HCC.

## INTRODUCTION

Human hepatocellular carcinoma (HCC) is currently the fifth most common cancer and third leading cause of cancer death worldwide [1]. HCC contains heterogeneous cell populations, and only a small subset of cells, termed cancer stem cells (CSCs), have the ability to drive and sustain tumor growth [2–5]. CSCs are endowed with stem cell properties such as the capability for extensive proliferation, self-renewal, differentiation into non-tumorigenic cancer cells and recapitulation of the original tumor in immunocompromised mice. Therefore CSCs are considered to be a pivotal target for tumor eradication.

Various stem cell markers are essential for identifying LCSCs. Previous studies have demonstrated that cluster of differentiation CD133 [6], CD90 [7], CD13 [8], CD24 [9], CD44 [10] and Epithelial cell adhesion molecule (EpCAM) [11] are cell surface markers for LCSCs. A multiple markers hypothesis has been suggested for CSCs in breast, pancreatic cancers and HCC [12, 13]. Yang *et al.* demonstrated that the CD90+CD44+ phenotype of liver CSCs may explain the aggressive growth pattern of HCC [7]. However, it remains unclear whether HCC patients with these markers share similar or distinct features, and whether combined detection of those markers would be more significant in predicting the prognosis of clinic-pathological characteristics in patients.

Understanding the pathways that regulate CSC self-renewal, differentiation and tumorigenicity may thus be critical to the development of effective anticancer therapies [14]. Developmental pathways such as Notch [15], Hedgehog [16] and Wnt/β-catenin [17–19] play important roles in normal stem cell function and are frequently altered in cancers. Notch activation promotes cell proliferation and the formation of stem cell-like colonies in human glioma cells [20], colon cancer [21] and breast cancer stem cells [22]. The Wnt/β-catenin pathway augments self-renewal capacity and inhibits the differentiation of colorectal and liver cancer stem cells [23–25]. We have previously demonstrated that Wnt/β-catenin signaling is downstream of the Notch pathway in regulating proliferation and malignant transformation of hepatic cell line L02/HBx [26]. However, recent studies reported that Notch is downstream of Wnt and negatively titrating active β-Catenin protein levels in stem/progenitor cells and colorectal cancer [27, 28]. As a result, it remains elusive whether Notch activity has a positive or negative effect on Wnt/β-catenin and how they affect each other in regulating the self-renewal of liver CSCs.

In this study, we found that simultaneous high expression of 4 different markers (CD90, CD24, CD13, CD133) correlates with poor prognosis in a total of 61 cases of HCC patients and serves as a promising predictor the prognosis of HCC patients. We also found that Notch and Wnt/β-catenin signaling pathways play a crucial role in maintaining the self-renewal of CD90, CD24, CD13, CD133 high expressed sphere-forming LCSCs. Notch1 may be downstream of Wnt/β-catenin signaling, and Notch1 negatively regulates Wnt/β-catenin signaling. There may also be a non-proteasome mediated feedback loop between those two signaling pathways.

## RESULTS

### 1 Expression of CD90, CD24, CD13 and CD133 in liver cancer cells correlates with poor prognosis in patients with HCC

To investigate whether cancer stem cell markers were over-expressed in HCC specimens, we retrospectively evaluated the expression levels of five cancer stem cell markers (CD90, CD44, CD133, CD13 and CD24) using IHC in 61 matched human HCC specimens and adjacent liver specimens. The markers CD90, CD44, CD133, CD13, and CD24 were present diversely in all HCC samples. By contrast, their expression in non-tumor (NT) liver tissues was almost absent (Supplementary Figure S1). The representative immunostaining of markers in tumor and uninvolved adjacent non-tumor tissues, and the pattern and intensity of staining for potential cancer stem cell markers in hepatocellular carcinoma specimens are shown in Supplementary Figure S1.

**Figure 1:**
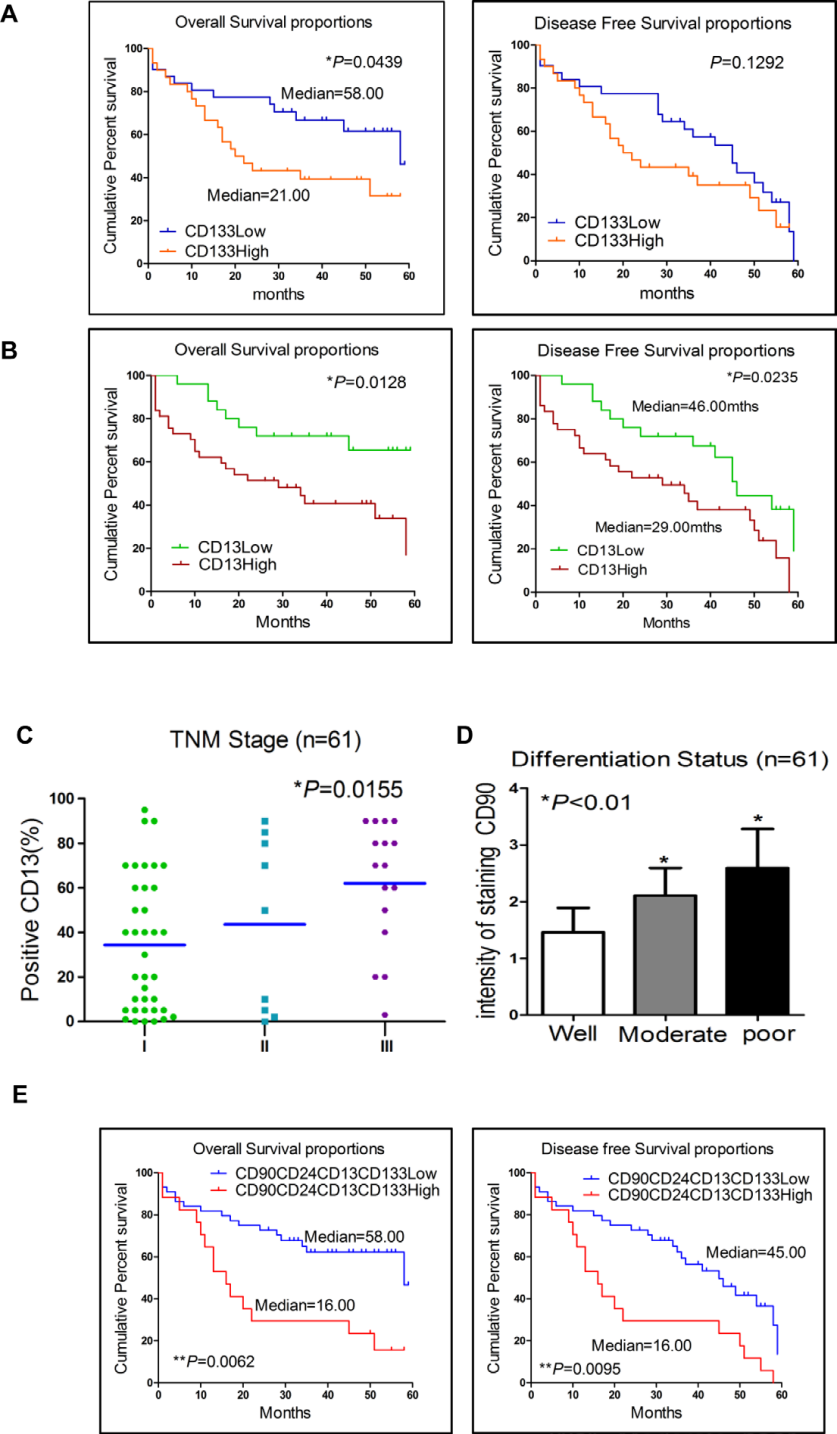
Expression of CD90, CD24, CD13 and CD133 in liver cancer cells correlated with poor prognosis in patients with HCC **A.** and **B.** Kaplan-Meier analyses for overall and disease-free survival were compared according to the CD133 and CD13 expression in tumor tissues. **C.** Patients who had higher advanced TNM Stages HCC (*p* = 0.0155, One-way ANOVA analysis) presented significantly higher CD13 expression. **D.** Patients with high CD90 expression had a significantly poorer differentiation status (*p* < 0.05, *t* test). **E.** Kaplan-Meier survival analysis of patients demonstrated that CD90CD24CD13CD133+ primary tumors displayed worse overall and disease-free survival. (log-rank test, *p* = 0.0062 and 0.0095).

Next, we investigated the clinical-pathologic correlation of those five markers expression. Our data showed that patients whose tumors over-expressed CD133 or CD13 had significantly shorter overall survival than those with lower CD133 or CD13 expression (*p* = 0.044 and *p* = 0.013, respectively, log-rank test, Figure 1A and 1B). Consistent with that finding, patients with CD133 or CD13 over-expression had shorter disease-free survival, though this finding with respect to CD133 did not reach statistical significance (*p* = 0.129 and *p* = 0.024, respectively, log-rank test). Patients whose tumors had significantly higher CD13 expression presented at more advanced TNM Stages (*p* = 0.016, One-way ANOVA analysis, Figure 1C) compared with their low CD13 expression counterparts. Patients with high CD90 expression also had significantly poorer differentiation status (*p* < 0.05, *t* test, Figure 1D). Univariate analyses of clinical pathologic correlations of all 5 markers expression in 61 HCC patients were shown in Supplementary Table S1. In summary, over-expression of LCSCs markers—CD90, CD44, CD133, CD13, and CD24—correlated with poorer differential status and shorter overall/disease-free survival.

Furthermore, we investigated the potential correlation between the expression of 4 different markers (CD90, CD24, CD13 and CD133) and the clinical outcomes of HCC patients. In a Kaplan-Meier survival analysis, patients with CD90CD24CD13CD133+/high primary tumors displayed worse overall and disease-free survival (estimated mean = 16 months) as compared to those patients with CD90CD24CD13CD133-/low primary tumors (estimated mean = 58 and 45 months, respectively, log-rank test, *p* = 0.0062 and 0.0095, respectively; Figure 1E). We also analyzed Kaplan-Meier survival, patients in stage I/II with CD90CD24CD13CD133+/high primary tumors displayed worse overall and disease-free survival as compared to those patients in stage I/II with CD90CD24CD13CD133-/low primary tumors (estimated mean = 54 and 13 months, respectively, log-rank test, *p* < 0.0001 and *p* = 0.0002, respectively, Supplementary Figure S2A). In terms of disease-free survival or overall survival, however, there were no statistical differences between other combinations of high expression and the low expression group, such as CD90CD44CD13CD133+/high, CD90CD44CD24CD133+/high and CD90CD44CD 13CD24+/high (data not shown). Our data showed that increased CD90CD24CD13CD133+/high expression in HCC not only correlated with advanced disease stage but also was associated with larger tumor size. Increased CD90CD24CD13CD133+ expression in HCC was more associated with higher tumor stages (TNM stages III or IV) than with lower tumor stages (TNM stages I or II) (*p* = 0.011; Table 1). The information of 17 patients with high CD90CD24CD13CD133 over-expression was shown in Supplementary Table S2. Furthermore, the logistic regression analysis showed that CD90CD24CD13CD133+ expression was inversely correlated with differentiation status in these 61 HCC patients (OR = 0.188, *Χ*^2^ test, *p* = 0.041, Table 1). Altogether, Over-expression of CD90, CD24, CD13 and CD133 in HCC correlated with more aggressive tumor behaviors and worse clinical outcomes in HCC patients.

**Table 1:** Multivariate analyses of clinico-pathologic correlation of CD90CD24CD13CD133 expression in 61 HCC patients

Clinico-Pathological		CD90CD24CD13CD133	
Variables	OR	95%CI	*P* value
Recurrence in the First Year (No/Yes)	1.09	0.11–10.7	0.9404
Portal venous Infiltration (Absence/Presence)	0.571	0.056–5.87	0.6378
TNM Stage (I/II/III)	0.123	0.024–0.619	0.0111*
Microsatellites (Absence/Presence)	–	–	0.9733
Differentiation Status (Well/Moderate/poor)	0.188	0.088–0.935	0.0412*
Tumor Size (< 5 cm/≥ 5 cm)	0.193	0.022–1.717	0.1402
No. of Tumor (1 / > 1)	0.102	0.017–0.600	0.0115*
HBV Association (Negative / Positive)	18	1.843–175.8	0.0129
HCV Association (Negative / Positive)	–	–	0.9657
Drinking (No/Yes)	2.222	0.232–21.26	0.4883
Chemotherapy (Absence/Presence)	–	–	0.9781
gross classification (massive/nodular/diffuse type)	1.379	0.198–9.604	0.7455
Serum AFP Level (≤ 20 ng/ml, > 20 ng/ml)	0.957	0.148–6.187	0.9633
Serum r-GT Level (≤ 40 U/l, > 40 U/l)	0.582	0.103–3.274	0.5390

* *P* < 0.05, Significant difference (logistic regression, Chi-Square test).

** *p* < 0.01, Significant difference (logistic regression, Chi-Square test).

### 2 Sphere-forming HCC cells possessed characteristics of cancer stem cells and the capacity to metastasize *in vivo* and *in vitro*

**Figure 2:**
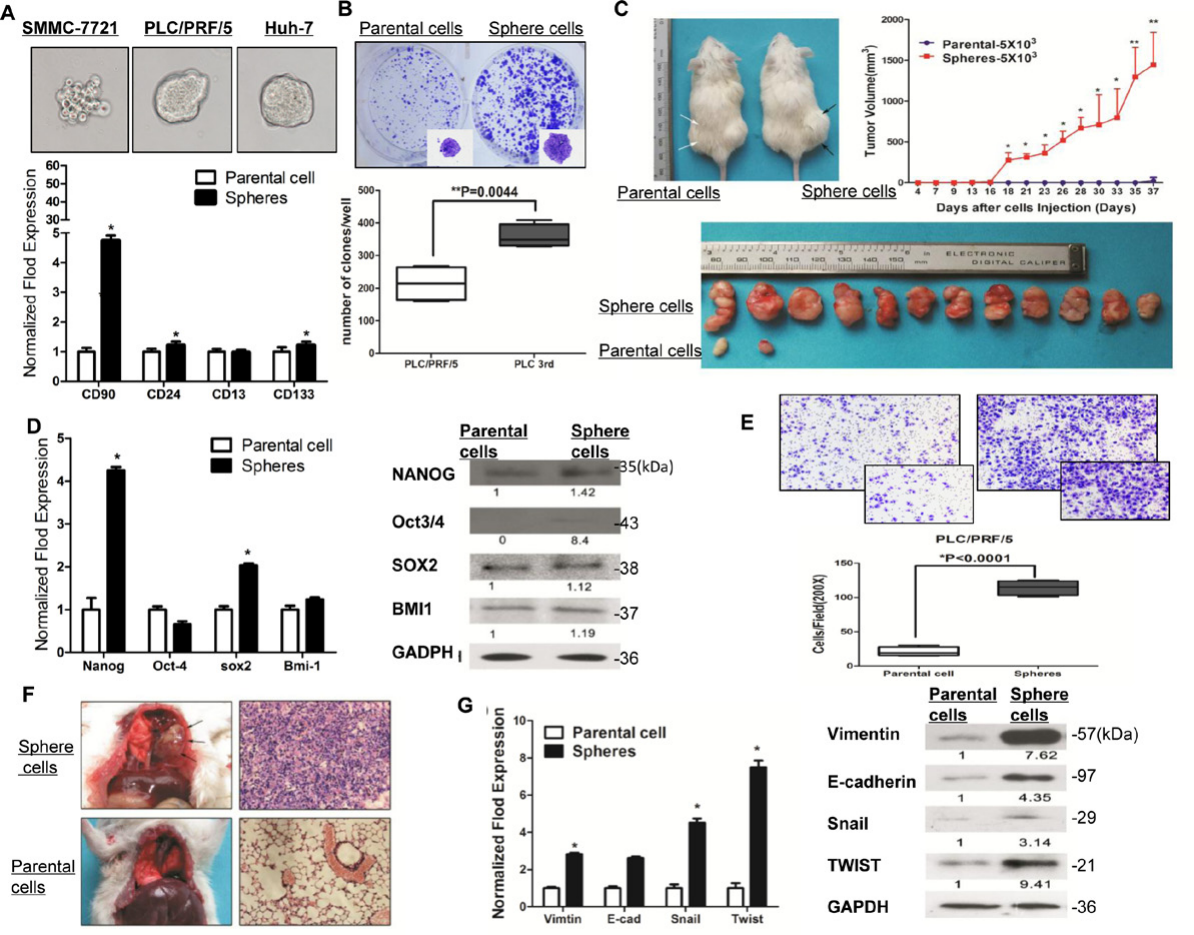
Sphere-forming HCC cells possessed characteristics of cancer stem cells and capacity to metastasize *in vivo* and *in vitro* **A.** The PLC/PRF/5 sphere-formation cells express higher cancer stem cells markers (CD90, CD24, CD13, CD133) (*p* < 0.05, *t* test). The representative spheres from HCC cell lines also shown on the top. **B.** The PLC/PRF/5 sphere-formation process higher colony formation efficiency (*p* = 0.044, *t* test). **C.** Efficiency of tumor formation and tumor volume of sphere-forming HCC cells (right black arrows) from PLC/PRF/5 and their parental cells (left white arrows) in NOD/SCID mice. **D.** RT-PCR and western blotting for the detection of stemness-associated genes over-expressed in PLC/PRF/5 sphere-formation cells. **E.** Transwell migration assay in parental cells and sphere-forming HCC cells (magnification times, 100X and 200X, respectively). **F.** Representative pulmonary metastasis of sphere-forming SMMC-7721 cells in NOD/SCID mice. Hematoxylin and eosin staining of a pulmonary metastasis tumor (right parts). **G.** The mRNA and protein expression level of EMT-related genes in sphere-forming SMCC-7721 cells.

We then enriched liver cancer stem cells in a three-dimensional sphere condition. We've analyzed the expression of all markers in 3rd passage of sphere-forming cells simultaneously by flow cytometry, and demonstrated that all the markers (CD90, CD24, CD133 and CD13) in sphere-forming cells were up-regulated. The expression of CD90, CD24, CD133 and CD13 were increased to 12.2%, 15.9%, 15.2% and 80.6% respectively, comparing to the parental cells, in which the expression of all markers are less than 2.6% (Supplementary Figure S2B). We also confirmed that the expression of CD90, CD24, CD13 and CD133 were approximately 26.7-fold, 6.9-fold, 2.6-fold and 8.8-fold higher respectively, compared with the parental cells. (*p* < 0.001, *t* test, Figure 2A and Supplementary Figure S3A).

Cancer stem cells (CSCs) are believed to possess the stem/progenitor properties of self-renewal [29], relative quiescence, tumorigenicity [30] and metastasis in immunodeficient mice [31]. In our study, colony formation assay revealed that sphere-forming cells derived from PLC/PRF/5 and Huh7 proliferated at a significantly higher rate than parental cells (*p* = 0.0044 and *p* = 0.0071, respectively, *t* test, Figure 2B and Supplementary Figure S3B). Additionally, to determine the tumorigenicity *in vivo*, sphere-forming HCC cells and parental cells were inoculated subcutaneously into NOD/SCID mice. Higher tumor incidence was observed in the sphere-forming HCC cells group (Figure 2C). Furthermore, as few as 1000 sphere-forming SMMC7721 (high potential of invasion and metastasis) and PLC/PRF/5 (comparatively low potential of invasion and metastasis) cells were sufficient for initial and consistent tumor development in NOD/SCID mice from 14d and 37d, respectively (Supplementary Table S3), which supports the greater self-renewal capability of sphere-forming HCC cells. Moreover, the expression of CD44, CD13 and CD24 is statistically higher in xenografts derived from sphere-forming PLC/PRF/5 and SMMC7721 cells, compared with the parental counterparts by IHC (Supplementary Figure S3C). Next, to determine whether sphere-forming HCC cells had other intrinsic properties of stem cells, we evaluated the expression of certain “stemness”-associated genes (NANOG, OCT3/4, SOX2 and BMI-1) [32–35] that are crucial in pathways and programs for establishing and maintaining stem cell-like characteristics. With qPCR and western blotting analysis, we found that CD90, CD24, CD13 and CD133 over-expressed fractions purified from PLC/PRF/5 cells had a general over-expression of these “stemness”-associated genes (Figure 2D).

To compare the relative quiescence of sphere-forming HCC cells and parental cells, cell cycle and apoptosis was measured by flow cytometry. We demonstrated that the proportion of sphere-forming PLC/PRF/5 cells in the G0/G1 phase was distinctly increased, while cells in the S and G2-M phase were significantly decreased, compared to the parental counterparts (*P* < 0.01, Supplementary Figure S3D). Furthermore, our results showed that the percentage of apoptotic cells in sphere-forming PLC/PRF/5 cells was significantly decreased (8.76 ± 0.39% versus 2.52 ± 0.35%, Supplementary Figure S3E), which means that sphere-forming cells induced cell cycle arrest and anti-apoptosis.

Vascular invasion is believed to be an important pathologic feature determining HCC metastasis and tumor recurrence [36, 37]. In our study, six out of eight (75%) patients with vascular invasion exhibited in CD90CD24CD13CD133+/high expression patients group (Supplementary Table S2), suggesting that CD90CD24CD13CD133+/high HCC cells were endowed with metastatic features. These data support the hypothesis that CD90CD24CD13CD133+/high CSCs represent a distinct invasive population that contributes to tumor metastasis. To test this hypothesis, we first isolated parental cells and sphere-forming cells from PLC/PRF/5 and examined their invasive abilities by using transwell migration assay. Compared to parental cells, sphere-forming cells displayed approximately 5.49-fold migration efficiency in transwell migration assay (*p* < 0.0001, *t* test, Figure 2E). To test the *in vivo* metastatic role of sphere-forming HCC cells, an experimental metastasis model was employed by injecting subcutaneously 5 × 10^5^ sphere-forming or parental SMMC-7721 cells into NOD/SCID mice. After 40 days, the formation of tumor foci in the lungs was evaluated with exploratory thoracotomy. 66% mice injected with sphere-forming SMMC-7721 cells showed tumor formation in the lungs, whereas none of the mice injected with parental cells showed tumor formation in the lungs (Figure 2F and Supplementary Table S3). Furthermore, the mRNA and protein expression levels of EMT-related genes and transcription factors Vimentin, Snail and Twist, significantly increased in sphere-forming PLC/PRF/5 cells (Figure 2G). Meanwhile, HCC cells with high CD90, CD24, CD44 or CD133 expression in patients' specimens significantly clustered around or in venous or lymphatic vessels invasion (Supplementary Figure S4A). Consistently, higher CD90CD24CD13CD133 expression group positively correlated with worse TNM Stage significantly (Table 1). Taken together, sphere-forming HCC cells expressed stem cell-associated genes, possessed the stem/progenitor properties, including extensive proliferation, exhibited an increased potential to self renew and metastasis *in vitro* and *in vivo*.

### 3 Notch and Wnt/β-catenin signaling pathway components were up-regulated in sphere-forming liver cancer stem cells

**Figure 3:**
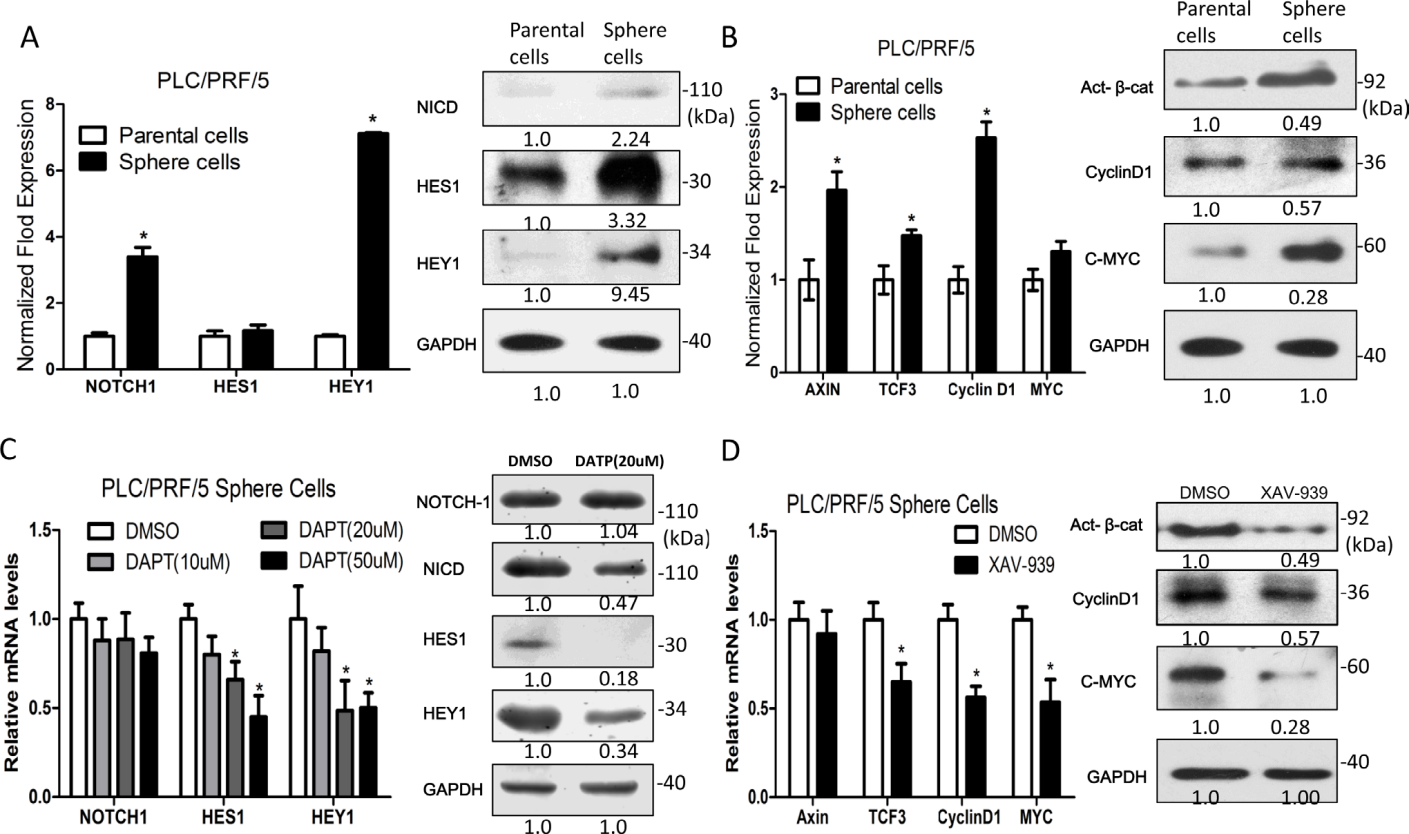
Notch and Wnt/β-catenin signaling pathway components are up-regulated in sphere-forming liver cancer stem cells **A.** and **B.** The mRNA and protein expression levels of Notch and Wnt/β-catenin signaling pathway components are up-regulated in sphere-forming liver cancer stem cells. **C.** and **D.** Notch and Wnt/β-catenin signaling pathway were inhibited by 20 μM of DAPT and 20 μM XAV939, respetctively.

We found that the high expression of Notch1 in HCC clinical specimens is associated with venous infiltration and poor prognosis (Data not shown). To investigate the molecular mechanisms involved in self-renewal and metastasis activity of sphere-forming LCSCs, the mRNA and protein expression levels of Notch signaling pathway components (NOTCH1, HES1 and HEY1) and Wnt/β-catenin signaling pathway components (Axin2, TCF3, CyclinD1 and MYC) were assessed. We found that the Notch and Wnt/β-catenin signaling pathway components are up-regulated in sphere-forming LCSCs HCC cells, compared to the parental counterparts (Figure 3A and 3B). To further demonstrate the biological function of Notch and Wnt/β-catenin signaling pathway in the self-renewal activity of sphere-forming LCSCs, we used a Notch-specific inhibitor, the γ-secretase inhibitor (DAPT) and a Wnt/β-catenin-specific inhibitor, tankyrase1/2 inhibitor (XAV939), to block the function of the Notch and Wnt/β-catenin signaling pathway, respectively (Figure 3C and 3D).

### 4 Notch and Wnt/β-catenin signaling pathways promoted stem-ness characteristics and metastasis potential in sphere-forming liver cancer stem cells

**Figure 4:**
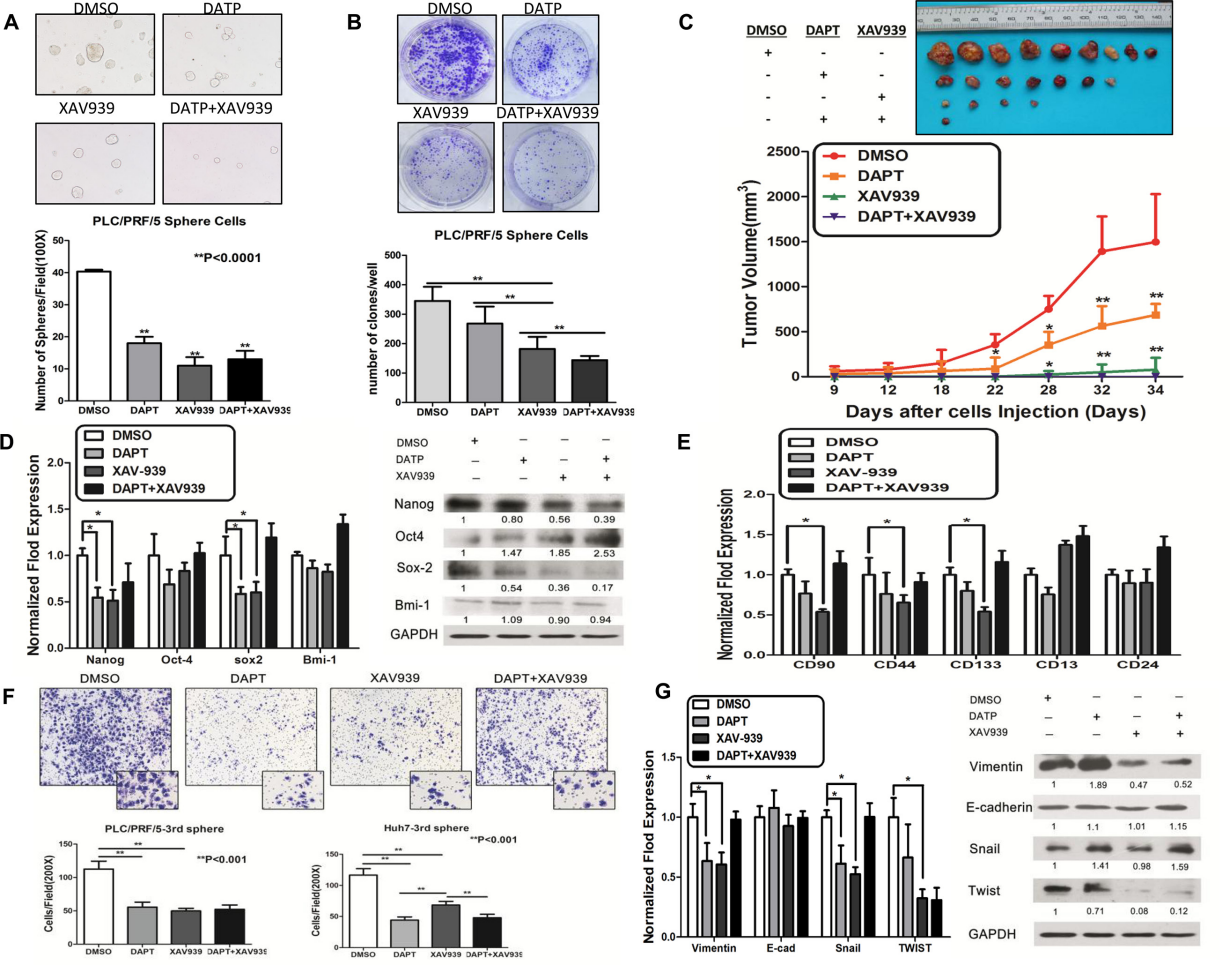
Notch and Wnt/β-catenin signaling pathways promoted stem-ness characteristics and metastasis potential in sphere-forming liver cancer stem cells **A.** and **B.** Sphere formation and colony formation ability (A) and efficiency of tumor formation (B) in sphere-forming PLC/PRF/5 decreased when inhibiting Notch and Wnt/β-catenin signaling pathway by 20 μM of DAPT and 20 μM XAV939. **C.** Efficiency of tumor formation and tumor volume of sphere-forming LCSCs were decreased by the blocking of Notch and Wnt/β-catenin. **D.** Stemness-associated genes (NANOG and SOX2) were significantly down-regulated upon Notch or/and Wnt/β-catenin inhibited (*p* < 0.05, *t* test). **E.** The cancer stem cells surface markers phenotype, CD90, CD44, CD133, were diminished by DAPT or XAV-939. But the decrease observed with DATP and XAV939 combined was no more than that observed by either individual treatment. **F.** and **G.** The metastasis capacity in transwell assay and EMT-related genes (Vimentin, Snail and Twist) were decreased in sphere-forming PLC/PRF/5 after inhibition of Notch and Wnt/β-catenin signaling pathway. Error bars represent standard deviation (SD) from at least three independent experiments.

To investigate further the roles of Notch and Wnt/β-catenin signaling pathways in maintaining the stem-ness characteristics and metastasis ability in sphere-forming LCSCs, we performed a sphere formation and colony formation assay. We demonstrated that DAPT, XAV939 and DAPT + XAV939 together could significantly inhibit sphere formation and colony formation in sphere-forming LCSCs derived from PLC/PRF/5 cells (Figure 4A and 4B). Furthermore, sphere-forming LCSCs derived from PLC/PRF/5 cells were subcutaneously inoculated into NOD/SCID mice, when the tumors reached 4 mm in diameter, the mice were injected intratumorally with DAPT, XAV939 and DAPT + XAV939 (Supplementary Table S4). We observed that both DAPT and XAV939 together could significantly inhibit the initiation and consistent tumor development in NOD/SCID mice after injected intratumorally in mice (Figure 4C and Supplementary Figure S4B). Additionally, we demonstrated that inhibition of DAPT, XAV939 and DAPT+XAV939 together could obviously attenuate the expression of the Notch intracellular domain (NICD) and β-catenin *in vivo* using IHC (Supplementary Figure S4B). Taken together, these results suggested that both Notch and Wnt/β-catenin signaling pathway play important roles in the regulation of self-renewal and the tumorigenicity of sphere-forming LCSCs. Consistently, upon Notch or/and Wnt/β-catenin inhibited, stemness-associated genes (NANOG and SOX2) were significantly down-regulated (Figure 4D, *p < 0.05, *t* test). Furthermore, our data demonstrated that the cancer stem cells surface markers phenotype, CD90, CD44 and CD133 decreased when Wnt/β-catenin and Notch were blocked (Figure 4E), indicating that Notch or/and Wnt/β-catenin blocking resulted in a differentiation of sphere-forming LCSCs. In addition, the down-regulation of Notch or/and Wnt/β-catenin with DAPT or/and XAV939 in sphere-forming LCSCs could significantly inhibit cell migration (Figure 4F). Inhibition of Notch or/and Wnt/β-catenin with DAPT or/and XAV939 could decrease the expression of EMT-associated transcription factors, Vimentin, SNAIL1 and TWIST1 (Figure 4G, *p < 0.05, *t* test). Collectively, these results suggested that both Notch and Wnt/β-catenin were essential factors for maintaining the self-renewal and metastasis of LCSCs.

### 5 Notch1 was downstream of Wnt/β-catenin signaling in liver cancer stem cells

**Figure 5:**
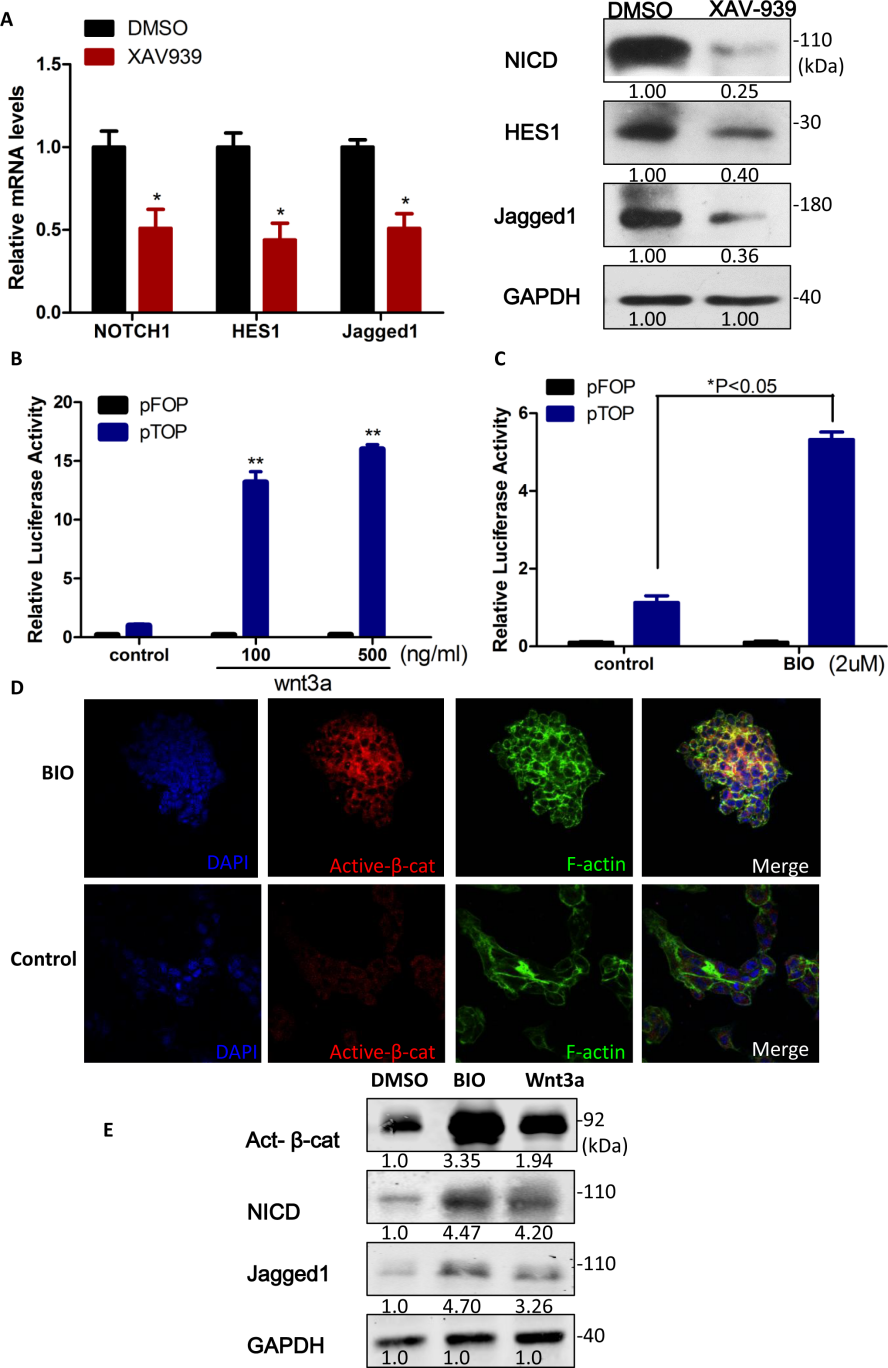
Notch1 was downstream of Wnt/β-catenin signaling in liver cancer stem cells **A.** Notch1 signaling pathway was dramatically down-regulated in set of the inhibition of Wnt/β-catenin by XAV939. **B.** and **C.** Relative β-catenin/TCF luciferase activity of sphere-forming LCSCs increased depending on the dosage of Wnt3a and BIO. **D.** IF Staining of Active β-catenin accumulated in sphere-forming LCSCs when treat with BIO (2 μM). **E.** Notch1 signaling pathway was up-regulated upon the activation of Wnt/β-catenin by Wnt3a (100 ng/ml) and BIO (2 μM). All luciferase values were normalized to Renilla activity (mean ± SD; *P* < 0:01). *P* values were determined using a two-tailed Student *t*-test, type II (see Materials and Methods). Gapdh antibody was used as a loading control. Numbers on western blots correspond to relative quantification.

Intriguingly, it has been demonstrated that Notch is downstream of Wnt in colorectal cancer cells through β-catenin-mediated transcriptional activation of the Notch-ligand Jagged1 [28]. To study the Notch and Wnt/β-catenin signaling pathway in sphere-forming liver cancer stem cells, we first employed XAV939 to block Wnt/β-catenin pathway. And the Notch signaling pathway components (NICD, Hes1 and Jagged1) were dramatically down-regulated 0.51-fold, 0.43-fold and 0.50-fold, respectively (*p* < 0.05, Figure 5A) in set of the inhibition of Wnt/β-catenin by XAV939. On the contrary, indeed activation of Wnt/β-catenin signaling by Wnt ligand Wnt3a or 6-bromoindirubin-3â€²-oxime (BIO), specifically inhibits GSK3 activity and inactivates the destruction complex, resulted in the dose-dependent increase in TCF/β-catenin-dependent transcriptional activity (Figure 5B and 5C, respectively), accumulation of active β-catenin (Figure 5D), NICD and Jagged1 (Figure 5E). Taken together, Notch1 positively correlated with the activation of Wnt/β-catenin, suggesting that Notch1 may be downstream of Wnt/β-catenin signaling in sphere-forming liver cancer stem cells.

### 6 β-catenin protein levels were diminished by Notch1 in liver cancer stem cells

**Figure 6:**
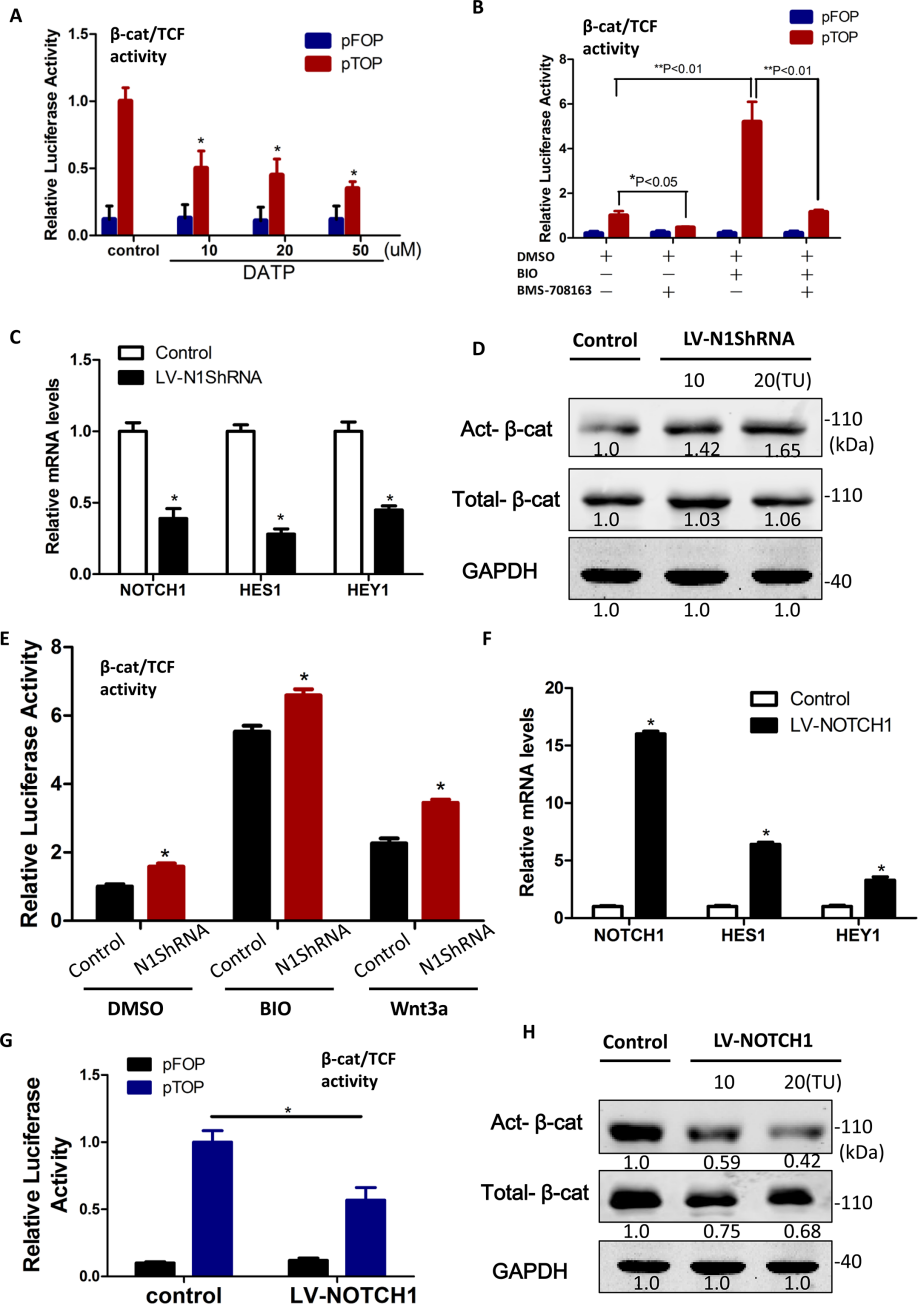
β-catenin protein levels were diminished by Notch1 in liver cancer stem cells **A.** Relative β-catenin/TCF luciferase activity of LCSCs decreased when treated with increasing doses of DAPT for 48 h. **B.** The activation of Wnt/β-catenin signaling by BIO (2 μM) rescue γ-secretase inhibitor (BMS-708163)-induced suppression of β-catenin/TCF dependent luciferase activity. **C.** Knockdown of Notch1 by LV-N1ShRNA. **D.** Reduced Notch1 levels which knockout by LV-N1ShRNA increased the transcriptionally active form of β-catenin protein. **E.** Relative β-catenin/TCF luciferase activity of sphere-forming LCSCs transfected with the control or LV-N1ShRNA in the presence or absence of BIO (2 μM) or Wnt3a (100 ng/ml; mean ± SD; *P* < 0:01). **F.** Notch1 was activited by LV-Notch1 after infected 48 h. **G.** Relative β-catenin/TCF luciferase activity of sphere-forming LCSCs transfected with the control or actived Notch1 (NICD) construct, LV-Notch1. **H.** active β-catenin protein levels increased after the over-expression of NICD by LV-Notch1.

To determine if Notch also regulates β-catenin protein levels in sphere-forming LCSCs, we blocked Notch endoproteolysis, which is mediated by the presenilin γ-secretase complex by DAPT, and investigated the regulation of β-catenin. Unexpectedly, we found that LCSCs treated with the γ-secretase inhibitor (GSI), DAPT, had a significant reduction of active β-catenin activity and protein levels in a dose-dependent fashion (Figure 6A). Moreover, the activation of Wnt/β-catenin signaling by BIO (2 uM) or Wnt3a (100 ng/ml) can rescue γ-secretase inhibitor (BMS-708163)-induced suppression of β-catenin dependent luciferase activity (Figure 6B and Supplementary Figure S4C). Conversely, the reduced Notch1 levels which were knocked out by lentiviral vector–mediated RNAi, LV-N1ShRNA (Figure 6C), did not affect the levels of total β-catenin protein but resulted in an increase in the dephosphorylated, transcriptionally active form of β-catenin protein (Figure 6D). We also found that LV-N1ShRNA LCSC exhibited significantly lower β-catenin/TCF dependent luciferase activity than controls, when stimulated with Wnt3a or BIO (Figure 6E). Finally, to confirm the Notch negatively regulation on β-catenin protein levels in LCSCs, we used lentiviral particles express NICD (Lv-Notch1) to over-express the cleavaged active-Notch1 levels (Figure 6F) in LCSCs and found a prominent decrease in β-catenin/TCF dependent luciferase activity (Figure 6G) and active β-catenin protein levels (Figure 6H). These results indicated that Notch1 negatively contributes to Wnt/β-catenin signaling modulation and is probably not proteasome mediated, which supports the earlier evidence showing Numb dependence and potential involvement of the lysosome [27].

## DISCUSSION

There is emerging evidence that HCC is driven and maintained by LCSCs that display stem cell properties. Therefore, tracing and “destemming” CSCs may be an effective strategy for treating HCC and improving patient outcomes.

In this study, we found that HCC patients with different CSC markers (CD133, CD90, CD13, CD24 or CD44) possessed distinct clinic-pathological features, suggesting that these cells with different cancer stem cell markers may be present in an identical HCC population. Furthermore, we have demonstrated that increased CD90CD24CD13CD133 expression in HCC not only correlates with advanced disease stage but also with larger tumor size and worse overall survival. This correlation suggests that CD90CD24CD13CD133+/high tumor cells have the potential to re-establish tumor growth in patients. Recently, several studies have also reported that successive passages of sphere-forming cells derived from mammary carcinoma cell line [38], pancreatic cancers [30] and cervical cancer [39, 40], displayed progressive cancer stem cells enrichment and different markers (CD90 and CD133) over-expression. Therefore, we hypothesized that liver cancer stem cells might concomitantly express two or more LCSCs markers instead of only one LCSCs marker.

To determine whether sphere-forming (CD90CD24CD13CD133+/high) HCC cells were LCSCs, we detected the stem-ness associated characteristics. Our data demonstrated that the sphere-forming HCC cells possessed progressively increasing self-renewal and tumor-initiating ability *in vitro* and *in vivo*. Furthermore, we found that CD90, CD24, CD44 or CD133 over-expression was also positively correlated with vascular infiltration, which is an important clinic-pathologic feature of HCC metastasis. The identification of sphere-forming HCC cells as a subpopulation involved in the CSC self-renewal of HCC might open up new perspectives for treatment.

Intriguingly, increasing evidence has shown that both Notch and Wnt signaling may play critical roles in the self-renewal of different CSCs [27, 41]. In particular, Notch signaling promotes the formation of cancer stem cells in human glioma [20] and inhibition of Notch signaling reduces primary tumor side population in breast cancer stem cells [42]; similarly, activation of Wnt1 signaling accelerates the proliferation rate and spheroids formation of gastric CSCs [43], while silencing of β-catenin by small interfering RNA could synergize the inhibition of self-renewal of LCSCs induced by BrMC [44]. Consistently, we found increased Notch and Wnt/β-catenin signaling pathway expression in the sphere-forming HCC cells. Inhibition of the Notch and Wnt/β-catenin signaling pathways each significantly attenuated sphere formation, colony formation, tumor development, and metastasis to the lungs capacity in NOD/SCID mice. This result suggests that both pathways play important roles in tumor formation and metastasis capacity. Furthermore, the decrease in cancer stem cells surface markers phenotype (CD90, CD44, CD133, CD13 and CD24) observed in combined DATP and XAV939 was no more than that observed by either individual treatment, suggesting that Notch and Wnt/β-Catenin may have cross-talk between each other.

Notch and Wnt/β-Catenin signaling often intersect in stem and progenitor cells and regulate one another transcriptionally [45]. The effects between each other are highly controversial. *Chulan et al.* recently demonstrated that Notch1 antagonizes Wnt/β-Catenin signaling by reducing levels of active β-Catenin in cardiac progenitor cells (CPCs) [27]. Conversely, the oncogenic effect of Notch1 on primary melanoma cells was mediated by β-catenin, which was upregulated following Notch1 activation [46]. This is in line with our previous study that Notch signaling is upstream of the Wnt pathway in regulating proliferation of L02/HBx cells [26]. In the present study, however, we discovered that Notch1 may be downstream of Wnt/β-catenin signaling. And Notch negatively regulates protein levels of active β-catenin in a post-translational manner in sphere-forming LCSCs. In our experiments, the interaction between these two critical regulatory proteins did not require ligand-dependent cleavage of Notch. Thus, in the presence of Wnt/β-catenin signaling, Notch may serve to titrating active β-catenin levels to temper the proliferative state of expanding cells [27]. It is likely Notch functions as a governor to balance tumor cell proliferation and the maintenance of the CSC population by regulating the β-catenin pathway. Contradictory data existed in stem cells and liver cancer stem cells suggesting that the versatile effects of Notch signaling pathway often depends on the context and timing as cells progress through stages of differentiation.

**Figure 7:**
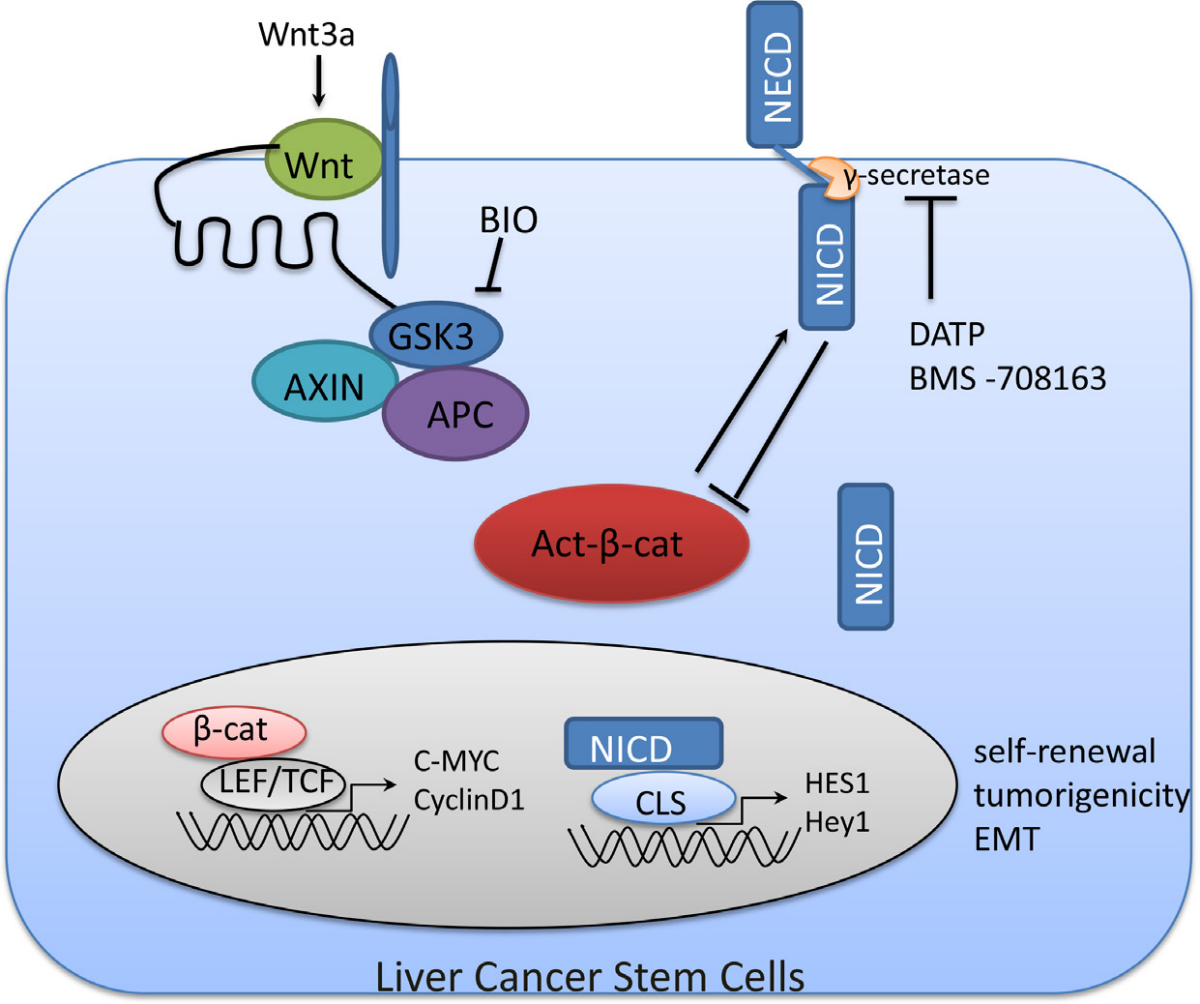
A non-proteasome mediated feedback loop between Notch1 and Wnt/β-catenin signaling in LCSCs The destruction complex in Wnt/β-catenin signaling is composed of Axin, APC and GSK3β. When the destruction complex of is inactivated by Wnt (Wnt3a) or BIO, dephosphorylated (active) β-catenin functions as a transcriptional activator with LEF/TCF. We show that Notch1 may be the downstream of Wnt/β-catenin. Active β-catenin protein levels can be negatively regulated by interaction with Notch. NECD, Notch extracellular domain; NICD, Notch intracellular domain.

In this study, we identified sphere-forming LCSCs (CD90CD24CD13CD133+/high) within these small lesions, and they functioned to initiate tumor growth and self-renewal through Notch and Wnt/β-catenin up-regulation. In addition, Notch1 was downstream of Wnt/β-catenin. There may be a non-proteasome mediated feedback loop between Notch1 and Wnt/β-catenin signaling in LCSCs (show in Figure 7). Although, the feedback loop between Notch1 and Wnt signaling need further study, the central role of Notch and Wnt/β-catenin signaling pathway in tumors may provide an attractive therapeutic strategy against HCC.

## MATERIALS AND METHODS

### Tissue collection and patient demographic information

Samples of paraffin-embedded sections of HCC and adjacent liver specimens were obtained from 61 HCC patients undergoing curative resection between 2008 and 2011 in Tongji hospital, Huazhong University of Science and Technology (HUST, Wuhan, China). Clinical data associated with those specimens was recorded without patient identification and all procedures were in accordance with the Huazhong University of Science and Technology Institutional Review Board protocols, and partial of human tissue samples. Informed consent was obtained from each subject. Patients were enrolled as described [47]. Survival data were determined at the last follow-up period for living patients. Tumor differentiation was defined according to the Edmondson grading system.

### Histology and immunohistochemistry

The IHC stained Samples from patients with primary antibodies Rabbit monoclonal anti-CD90/Thy1 (abcam, cat# ab92574), Rabbit Monoclonal anti-CD44 (ZSGE-BIO, cat# ZA-0537), Rabbit Polyclonal anti-CD24 (abgent, cat# # AP8782a), Mouse monoclonal anti-CD13 (ZSGE-BIO, cat# ZM-0284), Rabbit Polyclonal anti-CD133 (ZSGE-BIO, cat# ZA-0426) following the manufacturer. And the IHC stained tissue sections (Supplementary Materials and Methods) were analyzed individually by three pathologists without the patients' clinical characteristics. Staining for CD90, CD44, CD24, CD13 and CD133 was assessed using a relatively simple, reproducible scoring method. The intensity of staining was scored on a four point scale as negative (0), weak (1), medium (2) or strong (3). The extent of the staining, defined as the percentage of positive staining areas of tumor cells in relation to the whole tumor area, was scored on a scale of 0 to 4: 0 (0%), 1 (1–25%), 2 (26–50%), 3 (51–75%) and 4 (76–100%), see [48]. An overall protein expression score (overall score range, 0–12) was calculated by multiplying the intensity and positivity scores as described previously [49]. For statistical analysis, the final score was the mean value of scores from three observers. Scores ≤ 4 were considered as low expression, whereas scores ≥ 5 were considered as high expression. For statistical analysis, the final score was the mean value of scores from three observers. Examples of these are shown in Supplementary Figure S1.

### Cell lines and sphere culture

Human HCC cell lines (PLC/PRF/5, Huh7 and SMMC-7721) were obtained from American Type of Culture Collection (ATCC) and Cell Bank of Chinese Academy of Sciences (Shanghai, China). All of the cells were cultured as described [50]. For spheroid culture, cells were collected and washed to remove serum, then suspended in serum-free DMEM/F12 medium (cat#12400–024, GIBCO, Grand Island, NY) with B27 supplement (cat#17504–044; GIBCO, Grand Island, NY), 100 IU/ml penicillin, 100 μg/ml streptomycin, 20 ng/ml human recombinant epidermal growth factor (EGF, cat#PHG0311; GIBCO), 10 ng/ml human recombinant basic fibroblast growth factor (bFGF, cat#PHG0266; GIBCO), 2% B27 supplement without vitamin A, 1% N-2 supplement (cat#17502–048; GIBCO, Carlsbad, CA, USA) and 1% methyl cellulose(cat#M0262; Sigma-Aldrich) preventing cell aggregation. The cells were subsequently cultured in 100 mm ultra-low attachment dishes (cat#3262, Corning Life Sciences, Oneonta, NY, USA) at a density of 10^4^ cells/10 ml.

### Lentiviral-based transfection into HCC cells

For suppression or inhibition of Notch in HCC cells, lentiviral particles (Genechem, Shanghai) expressing Notch1-siRNA or NICD were used to regulate Notch signaling in sorted HCC cells. The siRNA sequence targeted Notch1 was listed as follows: 5â€²-GGAGCATGTGTAACATCAA-3â€². For optimization of transfection conditions with lentiviral vectors, HCC cells were infected with Lv-NICD or Lv-Notch1-si at different of multiplicity of infection (MOI) for 12 hours in the presence of 5 μg/ml of polybrene. Two days after infection, expression of green florescence protein was measured by FACS analysis. Infection of cells at MOI of 10 resulted in more than 90% of efficiency of infection without damaging cells (not shown in data).

### Luciferase reporter assay

For the TCF activity assay, pSUPER8 × TOPFlash or pSUPER8xFOPFlash (Addgene) and Renilla plasmids, kindly provided by Dr. Timothy R. Billiar (From the Department of Surgery, University of Pittsburgh School of Medicine, Pittsburgh), were co-transfected into HCC cells. Twenty-four hours after transfection, the cells were serum-starved for 24 h and stimulated with 1% FBS in DMEM. Luciferase activity was measured at 24 h after stimulation unless indicated using the Luciferase Assay System (Promega, Madison, MI). The assay was normalized with renilla as a transfection efficiency control.

### Statistical analysis

Statistical analysis and graphical presentation were performed using SPSS (v19.0) software for Windows (SPSS Inc., Chicago, IL). The logistic regression model was utilized to analyze the clinic-pathological parameters which were compared with log-rank test. The Cox regression model was used to perform univariate and multivariate analyses. The survival rate was calculated using the Kaplan–Meier method, and the resulting curves were compared by the log-rank test. *P* < 0.05 was considered significant. Other data in cell experiment are presented as mean ± standard deviation. When two groups were com-pared, the Student's *t* test was used. *P* < 0.05 was considered significant statistically and is marked with an asterisk. *P* < 0.01 was considered highly significant statistically and is marked with a double asterisk.

## SUPPLEMENTARY MATERIALS TABLES, FIGURES


