# Supplementary material for: Notch and Wnt/β-catenin signaling pathway play important roles in activating liver cancer stem cells
Source: Oncotarget. 2015 Dec 31;7(5):5754–68. doi: 10.18632/oncotarget.6805 (PMC4868719; doi:10.18632/oncotarget.6805)
Supplement: Supplementary file 1 [file oncotarget-07-5754-s001.pdf]

Notch and Wnt/β-catenin signaling pathway play important roles in activating liver cancer stem cells

Supplementary Materials

MATERIALS AND METHODS

DAPT, BMS-708163, XAV939, BIO and Wnt3a treatment

Cells were treated with DAPT (selleckchem, cat#S2215), BMS-708163 (selleckchem, cat#S1262), XAV939 (selleckchem, cat#S1180), BIO (selleckchem, cat#S7198) or Recombinant Human Wnt-3a Protein (R & D Systems, cat#5036-WN) at the indicated concentrations.

Sphere passage and sphere formation assay

The spheres were harvested by gentle centrifugation, then dissociated with trypsin-EDTA and mechanically disrupted with a pipette. The resulting single cells were then centrifuged to remove the enzyme and resuspended in serum-free medium above allowed to re-form spheres. The spheres should be passaged every 4–7 days before they reached a diameter of 100 µm. The dissociated single sphere-forming cells were also diluted to a density o f 500 cells/ml. Then, the 2 µl/well diluted cell suspension was plated to ultra low attachment 96-well plate (Corning Inc., Corning, NY, USA). The wells with only one cell were marked and observed every day. The size of spheres ware measured using program CellSens Standard Ink. (Olympus Inc., Center Valley, PA, USA).

Colony formation assay

The PLC/PRF/5 and Huh7 spheres were dissociated as described above. Trypan blue staining was used to determine cell viability, and more than 95% of cells with viability were acceptable for the following experiments. The single cells were seeded in DMEM with 10% FBS at a density of 2000 cells/well on 6-well plates that were pre-coated with Matrigel (BD Biosciences, San Jose, CA, USA). After 2 weeks, the colony formation ability was assessed by counting the number of colonies (> 70 cells) under a microscope after crystal violet staining (Sigma-Aldrich, St. Louis, MO, USA). Representative views were photographed. The parental cells were plated at the same density as the control.

Transwell migration assay

The invasion assay was performed using 24−transwell chambers (Costar, Bodenheim, Germany). Briefly, the parental adherent monolayer, tumor sphere SMMC-7721, PLC/PRF/5 and Huh7 cells or 20 uM DATP/15 uM XAV939 treated sphere cells were resuspended in serum−free DMEM. The upper chamber was loaded with 2 × 10^4cells/100 µl suspension and the lower chamber was loaded with 500 µl DMEM with 15% FBS. Following culture for 36 h, the cells in the upper chamber were removed and the lower chamber filter was fixed with 4% paraformaldehyde and stained with crystal violet. The number of cells that migrated to the undersurface of the membrane was counted and six randomly selected fields were analyzed. Three independent experiments were performed.

In vivo tumorigenicity experiments

All animal experiments were approved by the Institutional Animal Care and Use Committee of the Huazhong University of Science and Technology, which in according with the U.S. Public Health Service Policy on Use of Laboratory Animals. The PLC/PRF/5 parental and the third passage of sphere-forming cells with or without 10 µM DMSO, 20 µM DATP or 15 µM XAV939 pretreated for 7 days were used in tumorigenicity experiments. various numbers of viable single cells were subcutaneously injected into 5-week-old NOD/SCID male mice (animal centre of Tongji Medical College, Huazhong University of Science and Technology, Wuhan, China) in serum-free DMEM/Matrigel (1:1) using 100 µl microsyringe. Tumor-bearing mice and controls housed in the Animal Institute of Tongji Medical College in laminar flow cabinets for 45 days and sacrificed. The length (L) and width (W) of the tumors were measured externally with a vernier caliper every 3 days. Tumor volume was calculated with the formula: $V = (L \times W^2)/2$. Then the tumors were harvested for further examination.

Flow cytometry

We've analyzed the expression of all markers in 3rd passage of sphere-forming cells simultaneously by flow cytometry on IMM LSRII (Starzl Transplantation Institute, University of Pittsburgh School of Medicine, Pittsburgh, Pennsylvania). The antibodies are FITC Mouse Anti-Human CD90 (BD Pharmingen, #555595), PE Mouse Anti-Human CD24 (BD Pharmingen, #555428), PE-Cy™7 Mouse Anti-Human CD13(BD Pharmingen, #338425), CD133/1 (AC133) antibodies human (Miltenyi Biotec, #130098829).

RNA isolation, quantitative real-time PCR (qRT-PCR)

Total RNA was isolated from cultured cells using TRIzol reagent (Invitrogen) and cDNA was synthesized from 100 ng RNA using PrimeScript RT reagent Kit (Takara, Japan). Real-time quantitative PCR using SYBRP remix (TaKaRa) was performed as described previously [6]. Amplifications were performed in a Step One Real-Time PCR system (Applied Biosystems, USA) following the manufacturer's instructions. The expression of RNA was determined from the threshold cycle (Ct) and the relative expression levels were calculated by the 2-ΔΔCt method. All samples were assayed in triplicate. The primer sequences used to amplify specific target genes are listed in Supplementary Table S5.

Western blotting analysis and immunofluorescence

Quantified protein (40 μg) were separated on 8%–12% SDS-PAGE and were electrophoretically transferred onto nitrocellulose membranes (Millipore), which were blocked in PBST containing 5% milk for 2 hours at RT and immuno-blotted with the primary antibodies against full-length (FL)Notch1 (1:1000, CST), Cleaved Notch1 (Val1744,D3B8, CST), Hes1 (1:1000, CST), anti-Hey1 (1:600, Abcam), β-catenin (1:5000, SIGMA), active-β-Catenin (anti-ABC, 1:500, Millipore), anti-c-Myc (1:1000, CST), anti-CNND1 (1:800, Proteintech Group, Chicago, IL, USA), anti-Nanog (1:1000, CST), anti-Sox2,anti-Oct4,anti-E-cadherin (1:800, Proteintech Group), anti-vimentin (1:500, Proteintech Group), anti-Twist1 (1:800, Proteintech Group), anti-snail(1:1000, CST). The membranes were washed and incubated with horseradish peroxidase-labeled secondary antibody (1:5000; Santa Cruz Biotechnology), visualization was performed by an enhanced chemiluminescence kit (Pierce) and exposure to X-ray film (Kodak, Rochester, NY, USA). Immunoblotting with anti-GAPDH antibody was used as an internal control to confirme quivalent protein loading. Each experiment was repeated three times. Slides were viewed with Olympus Provismicroscope and FV1000 confocal microscope (Olympus).

Supplementary Table S1: Univariate analyses of clinico-pathologic correlation of 5 makers expression in 61 HCC patients

Clinico-Pathological	CD90		CD44		CD133		CD13		CD24	
**Variables**	OR	*P* value	OR	*P* value	OR	*P* value	OR	*P* value	OR	*P* value
**Recurrence in the First Year** (No/ Yes)	2.727	0.3024	2.000	0.3375	2.659	0.1426	1.944	0.2921	2.082	0.2524
**Portal venous Infiltration** (Absence/ Presence)	< 0.001	0.9713	5.833	0.0418	0.536	0.4238	0.173	0.1116	1.271	0.7610
**TNM Stage** (I/II/ III)	0.911	0.9208	1.409	0.5298	0.419	0.1075	0.227	**0.0140***	0.553	0.2964
**Microsatellites** (Absence/Presence)	< 0.001	0.9733	1.053	0.9674	2.000	0.5800	0.818	0.8729	4.444	0.2354
**Differentiation Status** (Well/Moderate/poor)	0.195	**0.0131***	0.565	0.2716	0.527	0.1968	0.231	**0.0073****	0.663	0.4323
**Tumor Size** (< 5 cm/≥ 5cm)	3.226	0.3084	4.667	0.0076	0.357	0.0891	0.400	0.0910	1.604	0.4021
**No. of Tumor** (1/>1)	2.000	0.4719	0.581	0.3633	0.468	0.2099	0.456	0.2283	0.604	0.4426
**HBV Association** (Negative/ Positive)	0.717	0.7821	6.500	0.1155	3.333	0.3096	0.676	0.7058	–	0.9578
**HCV Association** (Negative/ Positive)	4.364	0.3106	1.565	0.7556	0.439	0.5681	1.682	0.7180	2.105	0.6055
**Drinking** (No/Yes)	3.167	0.2282	1.500	0.4869	1.204	0.7531	0.833	0.7397	1.039	0.9474
**Chemotherapy** (Absence/Presence)	< 0.001	0.9781	1.565	0.7556	< 0.001	0.9534	< 0.001	0.9489	2.105	0.6055
**gross classification (massive/nodular/diffuse type)**	0.209	0.1139	0.272	0.1261	0.561	0.3981	0.985	0.9825	1.912	0.3941
**Serum AFP Level (≤ 20 ng/ml, > 20 ng/ml)**	3.000	0.3395	0.833	0.7397	1.211	0.7137	0.346	**0.0499***	1.062	0.9133
**Serum r-GT Level (≤ 40 U/l, > 40 U/l)**	0.971	0.9749	2.285	0.1346	0.607	0.3459	0.222	**0.0073****	0.380	0.0845

*$P < 0.05$, Significant difference (logistic regression, Chi-Square test).
**$P < 0.01$, Significant difference (logistic regression, Chi-Square test).

Supplementary Table S2: Characters of 17 patients with high CD90CD24CD13CD133 over-expression in all 61 patients

Patient No.	Age	Gender	Etiology	Survival time(m)	Recur. Time(m)	Venous Infiltration	TNM Stage	Diff.	Size (cm)	No. of Tumor	Drink	Chemo	AFP (ug/ml)	r-GT (U/l)
**P.20**	50	M	HBV	51	32 mo.	N	I	Moderate	6.4	1	N	N	189.79	56
**P.22**	58	M	HBV	13		Y	I	Moderate	9.7	1	N	N	14.91	90
**P.23**	53	F	Cryptogenic	10		N	I	poor	6.4	1	N	N	5.93	65
**P.25**	56	M	HBV	1		Y	III	poor	6.2	2	Y	N	820.36	107
**P.26**	53	M	Cryptogenic	5		N	II	poor	4.5	4	Y	N	58345	352
**P.31**	47	M	HBV	16		N	I	Moderate	10.5	1	Y	N	26724.86	67
**P.32**	68	M	HBV	1		N	I	Well	2.3	1	Y	N	18.18	112
**P.33**	50	M	HBV	22		Y	III	poor	5.5	1	N	N	11.69	87
**P.34**	38	M	HBV	45	28 mo.	Y	I	Well	2.5	1	N	N	15.55	78
**P.46**	40	F	HBV	58	4 mo.	N	III	Moderate	12.1	3	N	N	74424.48	–
**P.47**	44	F	HBV	55	49 mo.	N	III	poor	1.8	1	N	N	3.25	45
**P.49**	36	M	HBV	50	21 mo.	N	III	Moderate	4.9	1	Y	N	15.98	63
**P.50**	47	M	HBV	11		N	III	Moderate	6	1	N	N	76.39	90
**P.51**	51	M	HBV	9		Y	III	Moderate	1.5	1	N	N	7416.42	–
**P.52**	48	M	HBV	2		Y	III	Moderate	6.7	3	N	N	40621.91	274
**P.53**	28	M	HBV	13		N	II	poor	7.6	1	N	N	485.2	99
**P.56**	58	M	HBV	20		N	III	Moderate	7.5	4	1	N	4356.67	42
**P.59**	53	M	HBV	17	7 mo.	N	I	Moderate	8.2	1	0	N	1468.13	24

Supplementary Table S3: Tumorigenicity experiments of PLC/PRF/5 and SMMC-7721 sphere-forming cells and parental cells in NOD/SCID mice

Cell type	Cell numbers injected	Tumor incidence[1]	Latency (days)[2]	Distant organ metastasis[3]
**PLC/PRF/5**				
**Sphere-forming cells**	1×10^3	1/3	37	−
	5×10^3	3/3	26	−
	1×10^4	3/3	24	−
	5×10^4	2/3	18	−
**parental cells**	1×10^3	0/3	−	−
	5×10^3	0/3	−	−
	1×10^4	0/3	−	−
	5×10^4	0/3	−	−
**SMMC-7721**				
**Sphere-forming cells**	1×10^3	3/3	14	−
	5×10^3	3/3	12	−
	1×10^4	3/3	9	−
	5×10^4	3/3	14	−
	5×10^5	3/3	7	Yes (2/3)
**parental cells**	1×10^3	0/3	−	−
	5×10^3	1/3	37	−
	1×10^4	2/3	30	−
	5×10^4	2/3	18	−
	5×10^5	3/3	7	No (0/3)

1. No. of Mice with Tumor Formation/Total No. of Mice with Cell Injection.
2. Approximate No. of days from tumor cell injection to the first appearance of tumors.
3. Pulmonary metastasis testified by IHC.

Supplementary Table S4: Tumorigenicity experiments of PLC/PRF/5 sphere-forming cells treated with inhibitors of Notch1 and Wnt/β-catenin

Sphere-forming cells	Cell numbers injected	Tumor incidence[1]	Latency (days)[2]
**PLC/PRF/5**			
**DMSO**	5×10^3	3/3	9
	1×10^4	3/3	9
	5×10^4	3/3	9
**DAPT**			
	5×10^3	2/3	22
	1×10^4	2/3	22
	5×10^4	3/3	9
**XAV939**			
	5×10^3	1/3	32
	1×10^4	0/3	22
	5×10^4	1/3	28
**DAPT+XAV939**			
	5×10^3	0/3	−
	1×10^4	1/3	28
	5×10^4	0/3	−

1. No. of Mice with Tumor Formation/Total No. of Mice with Cell Injection.
2. Approximate No. of days from tumor cell inject ion to the first appearance of tumors.

Supplementary Table S5: Primer sequences used to amplify specific target genes

Gene Sequences		Sequences(5′–3′)	PCR product (bp)	GenBank accession no.
CD24	Sense	CTCCTACCCACGCAGATTTATTC	166	NM_001291737.1
	Antisense	AGAGTGAGACCACGAAGAGAC		
CD13(ANPEP)	Sense	TTCAACATCACGCTTATCCACC	169	NM_001150.2
	Antisense	AGTCGAACTCACTGACAATGAAG		
CD133(PROM1)	Sense	AGTCGGAAACTGGCAGATAGC	99	NM_001145848.1
	Antisense	GGTAGTGTTGTACTGGGCCAAT		
CD90(THY1)	Sense	ATCGCTCTCCTGCTAACAGTC	135	NM_006288.3
	Antisense	CTCGTACTGGATGGGTGAACT		
CD44	Sense	CTGCCGCTTTGCAGGTGTA	109	NM_000610.3
	Antisense	CATTGTGGGCAAGGTGCTATT		
Oct3/4(POU5F1)	Sense	CTTGAATCCCGAATGGAAAGGG	164	NM_001252452.1
	Antisense	GTGTATATCCCAGGGTGATCCTC		
SOX2	Sense	GCCCTGCAGTACAACTCCAT	128	NM_003106.3
	Antisense	GACTTGACCACCGAACCCAT		
NANOG	Sense	GTCCCAAAGGCAAACAACCC	108	NM_001297698.1
	Antisense	GCTGGGTGGAAGAGAACACA		
E-cadherin(CDH1)	Sense	ATTTTTCCCTCGACACCCGAT	109	NM_004360.3
	Antisense	TCCCAGGCGTAGACCAAGA		
Vimentin(VIM)	Sense	TGCCGTTGAAGCTGCTAACTA	248	NM_003380.3
	Antisense	CCAGAGGGAGTGAATCCAGATTA		
TWIST1	Sense	GTCCGCAGTCTTACGAGGAG	156	NM_000474.3
	Antisense	GCTTGAGGGTCTGAATCTTGCT		
NOTCH1	Sense	CCGCAGTTGTGCTCCTGAA-	109	NM_017617.3
	Antisense	ACCTTGGCGGTCTCGTAGCT		
HES1	Sense	GCTAAGGTGTTTGGAGGCT	122	NM_005524.3
	Antisense	CCGCTGTTGCTGGTGTA		
CyclinD1(CCND1)	Sense	GGCTGAAGTCACCTCTTGGTTACAG	177	NM_053056.2
	Antisense	TAGCGTATCGTAGGAGTGGGACAG		
MYC	Sense	AAGAGGACTTGTTGCGGAAA	179	NM_002467.4
	Antisense	CTCAGCCAAGGTTGTGAGGT		
BMI1	Sense	GCTGCCAATGGCTCTAATGAA	189	NM_005180.8
	Antisense	TGCTGGGCATCGTAAGTATCTT		
actin	Sense	GTTGCGTTACACCCTTTCTTG	157	NM_001017992.3
	Antisense	GACTGCTGTCACCTTCACCGT		

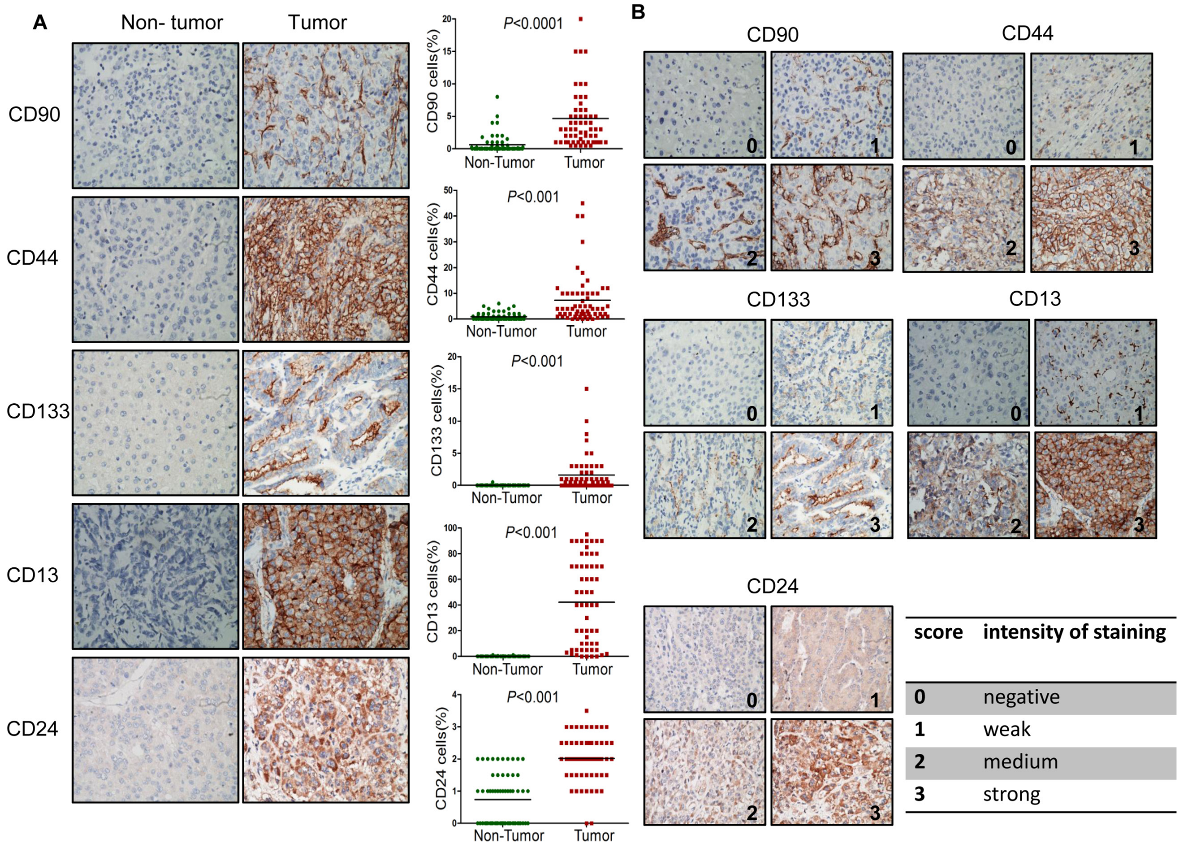

score	intensity of staining
0	negative
1	weak
2	medium
3	strong

Supplementary Figure S1: (**A**) Markers immunostaining in tumor and uninvolved adjacent non-tumor tissues. Sections were immunostained as described and comparison samples are shown of HCC and surrounding non-tumor liver from the same specimens for CD90, CD44, CD133, CD13 and CD24. Bar 400X. (**B**) Pattern and intensity of staining for potential cancer stem cell markers in hepatocellular carcinoma specimens. Formalin-fixed paraffin-embedded human HCC samples were immunostained for CD90, CD44, CD133, CD13 and CD24 and intensity of staining was assessed as described in methods. For each marker, example images are shown demonstrating the staining pattern for each of the intensity grades. Bar 400X.

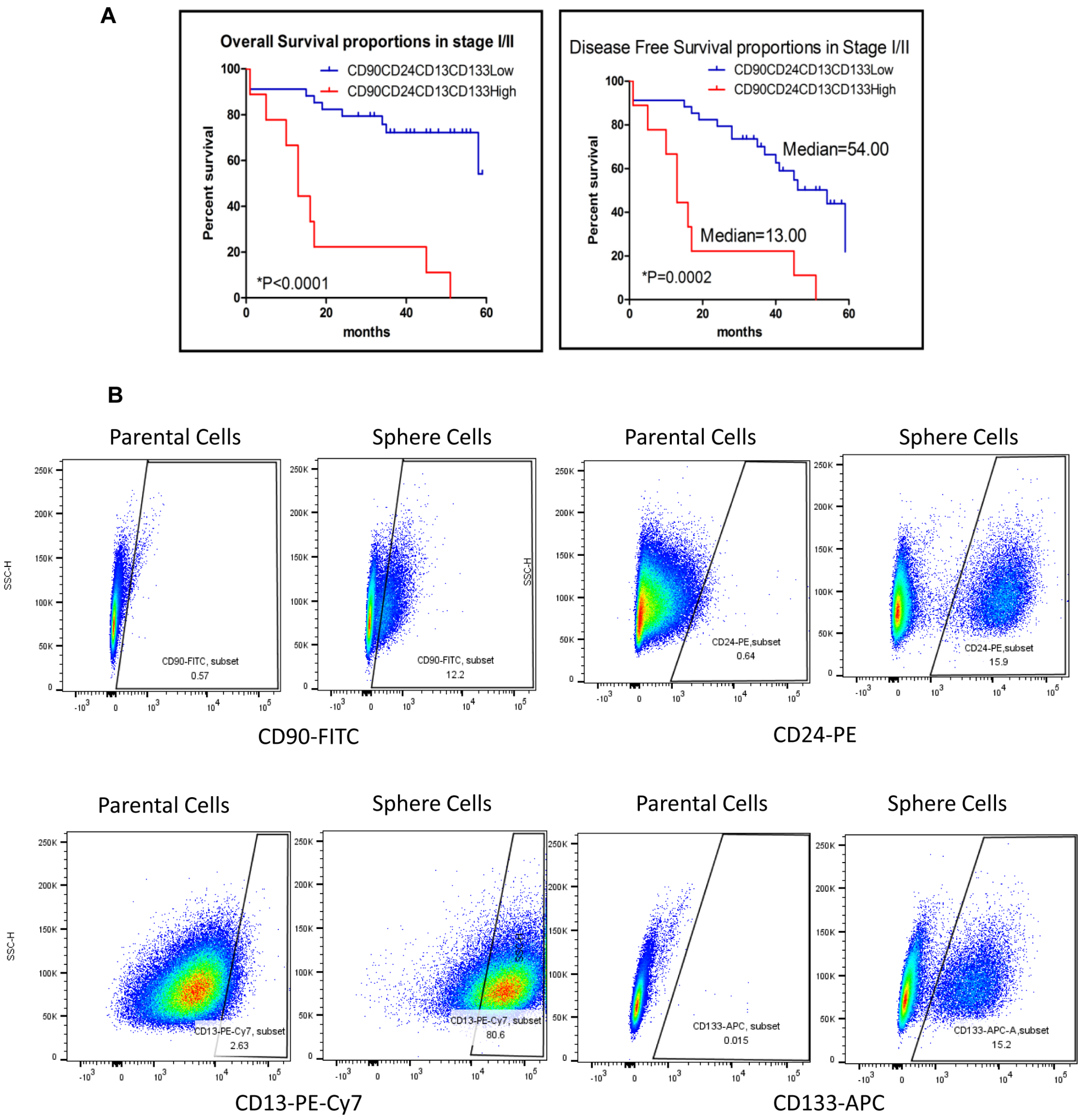

Supplementary Figure S2: (**A**) Kaplan-Meier survival, patients in stage I/II with CD90CD24CD13CD133+ primary tumors displayed worse overall and disease-free survival as compared to those patients in stage I/II with CD90CD24CD13CD133-/low primary tumors. (**B**) The expression of CD90+, CD24+, CD133+ and CD13+ were increased to 12.2%, 15.9%, 15.2% and 80.6% respectively, comparing to the parental cells, in which the expression of all markers are less than 2.6%.

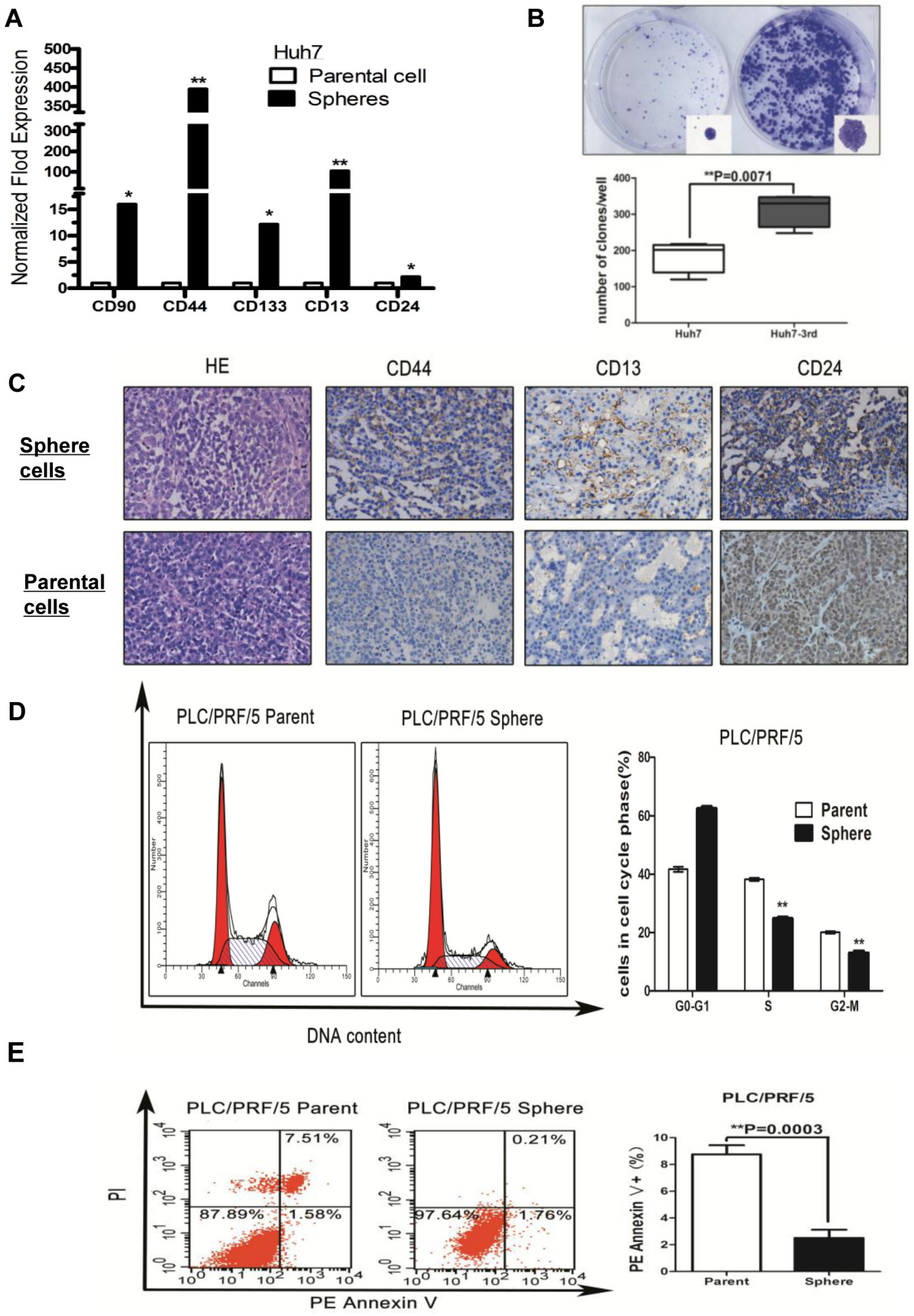

Supplementary Figure S3: (**A**) The cancer stem cells markers are up-regulated in sphere cells in Huh7. (**B**) Colony formation assay revealed that sphere-forming HCC cells derived from PLC/PRF/5 and Huh7 proliferated at a significantly higher rate than parental cells. (**C**) The expression of CD44, CD13 and CD24 is statistically higher in xenografts derived from sphere-forming PLC/PRF/5 cells, compared with the parental counterparts by IHC. (**D** and **E**) PLC/PRF/5 sphere-forming cells induced cell cycle arrest in G0/G1 phase and anti-apoptosis ($p < 0.05$, *t* test). Error bars represent standard deviation (SD) from at least three independent experiments.

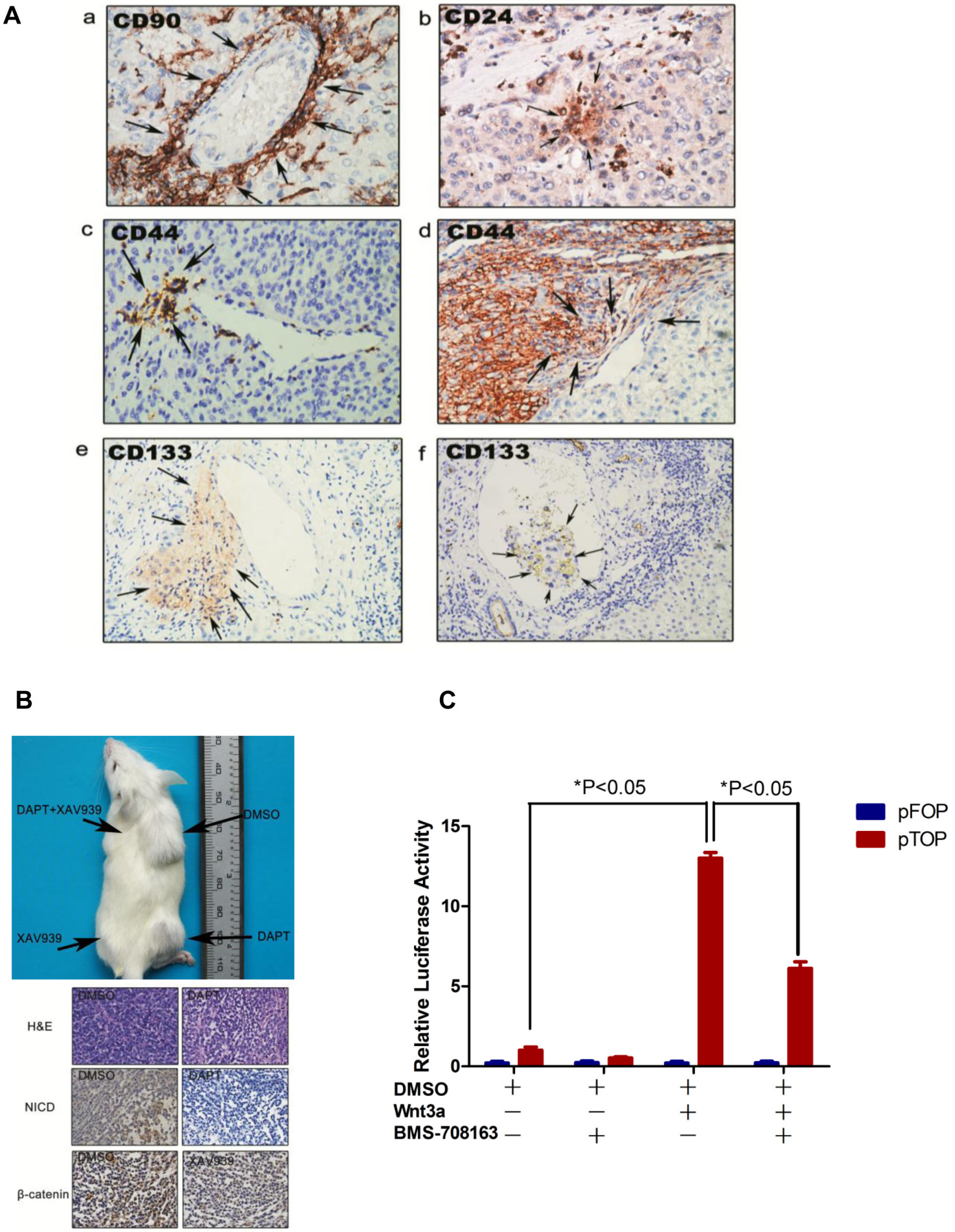

Supplementary Figure S4: (**A**) Representative microphotograph of CD90, CD24, CD44 and CD133 expression in HCC tissues by IHC analysis. Arrows indicate their expression clustered around or in venous or lymphatic vessels. (**B**) The representative NOD/SCID mice injected intratumorally with DMSO (20 μM), DAPT (20 μM), XAV939 (20 μM) and DAPT+XAV939 (upper panel). Immunohistochemistry of NICD and active-β-catenin expression in xenografts tissues from NOD/SCID mice above (bottom panel). (**C**) The activation of Wnt/β-catenin signaling by Wnt3a (100 ng/ml) rescue γ-secretase inhibitor (BMS-708163)-induced suppression of β-catenin/TCF dependent luciferase activity.
